# Assessment of the characteristics and biocompatibility of gelatin sponge scaffolds prepared by various crosslinking methods

**DOI:** 10.1038/s41598-018-20006-y

**Published:** 2018-01-25

**Authors:** Gang Yang, Zhenghua Xiao, Haiyan Long, Kunlong Ma, Junpeng Zhang, Xiaomei Ren, Jiang Zhang

**Affiliations:** 1 Department of Medical Information and Engineering, School of Electrical Engineering and Information, Sichuan University, Chengdu, 610065 China; 2 Department of Cardiovascular Surgery, West China Hospital, Sichuan University, Chengdu, 610041 China; 3 Center of Engineering-Training, Chengdu Aeronautic Polytechnic, Chengdu, 610100 China; 4 Department of Orthopaedics, Yongchuan Hospital, Chongqing Medical University, Chongqing, 402160 China

## Abstract

This comparative study aims to identify a biocompatible and effective crosslinker for preparing gelatin sponges. Glutaraldehyde (GTA), genipin (GP), 1-ethyl-3-(3-dimethyl aminopropyl)carbodiimide (EDC), and microbial transglutaminase (mTG) were used as crosslinking agents. The physical properties of the prepared samples were characterized, and material degradation was studied *in vitro* with various proteases and *in vivo* through subcutaneous implantation of the sponges in rats. Adipose-derived stromal stem cells (ADSCs) were cultured and inoculated onto the scaffolds to compare the cellular biocompatibility of the sponges. Cellular seeding efficiency and digestion time of the sponges were also evaluated. Cellular viability and proliferation in scaffolds were analyzed by fluorescence staining and MTT assay. All the samples exhibited high porosity, good swelling ratio, and hydrolysis properties; however, material strength, hydrolysis, and enzymolytic properties varied among the samples. GTA–sponge and GP–sponge possessed high compressive moduli, and EDC–sponge exhibited fast degradation performance. GTA and GP sponge implants exerted strong *in vivo* rejections, and the former showed poor cell growth. mTG–sponge exhibited the optimal comprehensive performance, with good porosity, compressive modulus, anti-degradation ability, and good biocompatibility. Hence, mTG–sponge can be used as a scaffold material for tissue engineering applications.

## Introduction

Gelatin is a partial hydrolysis product of native collagen and characterized by non-toxicity, non-carcinogenicity, biocompatibility, and biodegradability^1–3^; gelatin is widely used in the pharmaceutical and medical fields, such as in wound dressing materials^4,5^, tissue engineering scaffolds^6–8^, and drug delivery carriers^9,10^. Gelatin can be prepared in a spongy form to be suitable for tissue engineering applications. The porous 3D structure of gelatin sponge scaffolds can provide numerous spaces for cell adhesion. However, gelatin scaffolds exhibit weak mechanical strength and poor hydrolysis resistance. As such, gelatin scaffolds are stabilized by material crosslinking to increase their strength and hydrolysis resistance and maintain their stability during implantation^11^. Crosslinking agents are introduced into gelatin through physical methods, such as dehydrothermal^12,13^ and ultraviolet radiation treatment^14^; use of chemical agents, such as glutaraldehyde (GTA)^15,16^, carbodiimides^2,8^, and genipin (GP)^13,15^; and use of enzymes, such as transglutaminase^17–19^, tyrosinases^20^ and horseradish peroxidases^21^.

GTA is one of the most widely used crosslinking agents in the field of biomedicine because it can effectively stabilize collagen or its derivatives; however. GTA is cytotoxic^15^. Crosslinking of collagen or its derivatives with GTA involves reactions between the free amine groups of lysine or hydroxylysine amino acid residues in the collagenous polypeptide chains and the aldehyde groups of GTA to produce imine linkages^22^. GP is a natural substance extracted from geniposide, which is found in gardenia plants; GP exhibits relatively lower toxicity than GTA. GP reacts with amino acids in collagen or its derivatives containing amine side groups, such as lysine and arginine, to form a dark blue pigment, which is used in the manufacture of food dyes^15^. 1-Ethyl-3-(3-dimethyl aminopropyl)carbodiimide (EDC) is used for crosslinking of polysaccharides and proteins. EDC participates in the reaction among molecules containing free carboxylic and amine groups to form amide bonds.

Transglutaminase has received increasing attention because of its ability to crosslink proteins. This enzyme catalyzes acyl transfer reactions between the *λ*-carboxyamide groups of glutamine residues (acyl donor) and the *ε*-amino groups of lysine residues (acyl acceptor) to form ε-(*λ*-glutaminyl) lysine intra- and intermolecular crosslinked proteins^23^. In the past, the high cost of mammalian-derived transglutaminase limited its application. Currently, transglutaminase extracted from microorganisms exhibits high yield and low price and is thus suitable to be used as a crosslinking agent^24^. In our previous paper, we evaluated the effect of mTG as a crosslinker for preparing gelatin hydrogels and found that it exhibits good cell biocompatibility^25^. To the best of our knowledge, the use of mTG as a crosslinker in the preparation of gelatin sponge scaffolds has not been reported yet.

This comparative study aims to identify a biocompatible and effective crosslinker for preparing gelatin sponge scaffolds. GTA, GP, EDC, and mTG were used as crosslinking agents. The prepared samples were characterized using porosity measurement, compression modulus test, and water absorption analysis. The enzymolytic properties of the materials were also studied using various proteases. The prepared sponges were subcutaneously implanted in SD rats to assess their *in vivo* degradation behavior. Adipose-derived stromal stem cells (ADSCs) were cultured and inoculated onto the sponges to compare their cellular biocompatibility. Cell seeding efficiency and digestion time in the sponges were evaluated. Cellular viability and proliferation in the scaffolds were further analyzed by cellular fluorescent staining and MTT assay.

## Results

### Material appearance and physical characteristics

#### Material appearance

**Figure 1:**
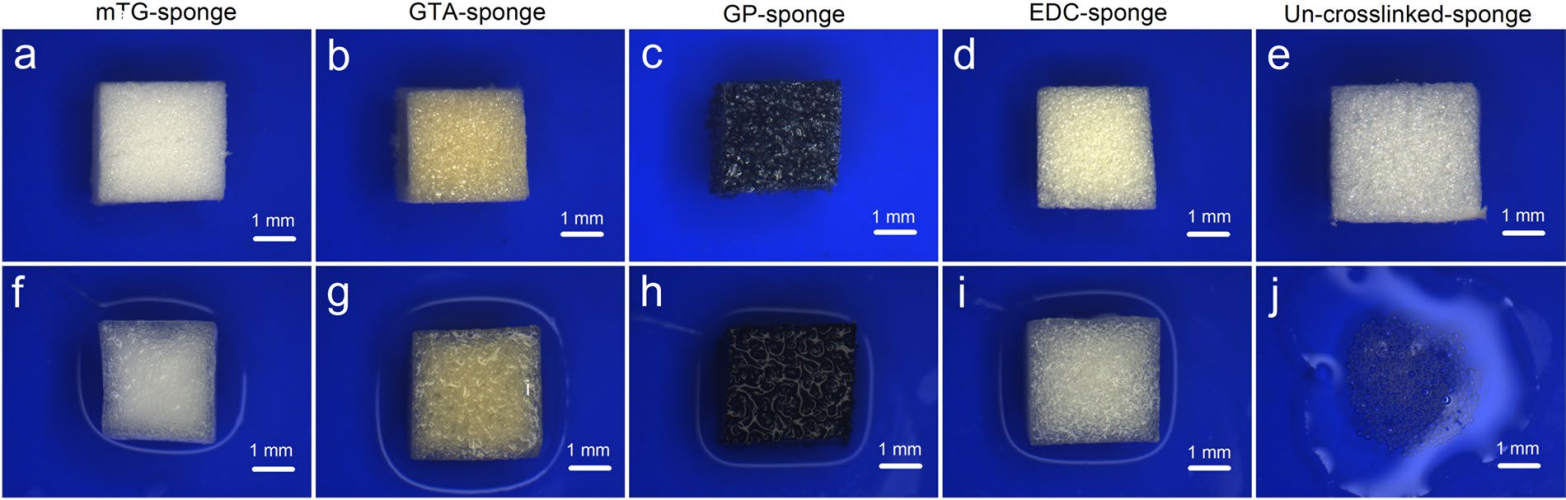
Material appearance of gelatin sponges prepared by different crosslinking methods. (**a**–**e**) In the dry state; and (**f**–**j**) in the wet state.

Gelatin sponges prepared by different crosslinking methods show various colors. mTG–sponge, EDC–sponge, and un-crosslinked gelatin sponge are white. GP–sponge is dark blue, and GTA–sponge is light yellow (Fig. 1). After absorbing water, the color of GP–sponge deepened, appearing black. The wet GTA–sponge is yellow, and the wet EDC–sponge and mTG–sponge are translucent. The un-crosslinked gelatin sponge was dissolved immediately once immersed in aqueous solution at 37 °C and thus cannot be used as a scaffold material.

#### Porosity analysis

**Figure 2:**
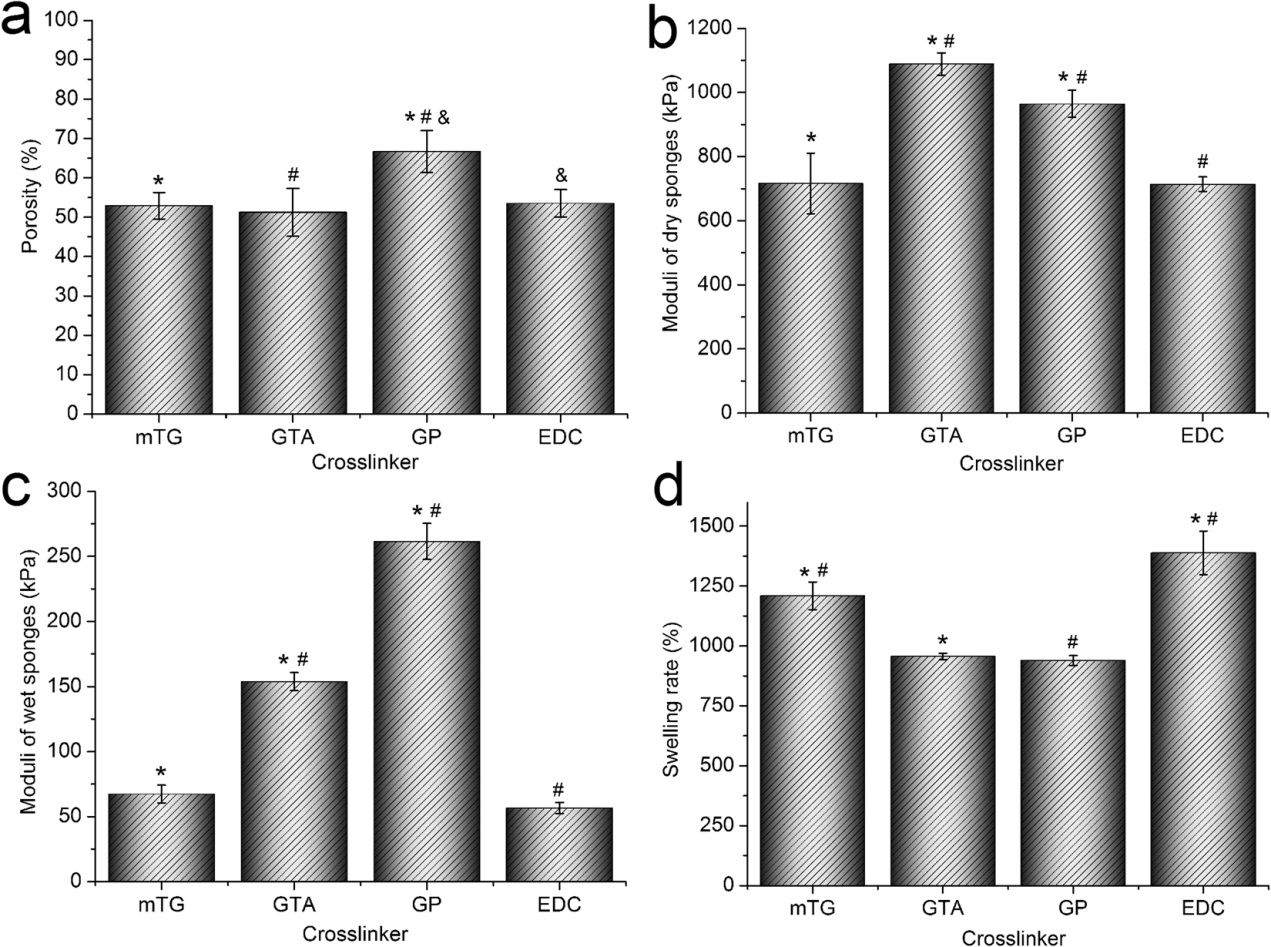
Physical characteristics of gelatin sponges prepared by different crosslinking methods. (**a**) Porosity; (**b**) compressive elastic modulus of dry gelatin sponges; (**c**) compressive elastic modulus of wet gelatin sponges; and (**d**) swelling ratio. ^*,#,&^*P* < 0.05.

The pore size of GP–sponge varies significantly, and the average porosity is 66.6% ± 5.3%, which is the largest among all sponges. The other three kinds of sponges show more uniform pore sizes and slightly smaller porosities than those of GP-sponge. The porosity levels of mTG–sponge and EDC–sponge are 52.9% ± 3.4% and 53.5% ± 3.5%, respectively. the porosity of GTA–sponge is the smallest, with a value of 51.2% ± 6.1% (Fig. 2a).

#### Compressive elastic modulus analysis

In the dry state, the elastic moduli of the four kinds of crosslinked gelatin sponges are relatively high. The elastic moduli of dry mTG–sponge and EDC–sponge are almost equal, with values of 716.2 ± 94.5 kPa and 714.2 ± 23.0 kPa, respectively. The modulus of dry GP–sponge is 964.2 ± 42.0 kPa, and that of GTA–sponge is the highest, with a value of 1088.6 ± 34.7 kPa (Fig. 2b). The elastic moduli of the sponges significantly decrease after absorbing water. The elastic moduli of wet mTG–sponge and EDC–sponge are 67.4 ± 6.8 kPa and 56.6 ± 4.2 kPa, respectively. The elastic modulus of wet GTA–sponge is 153.8 ± 6.9 kPa, and that of the wet GP–sponge is the highest, with a value of 261.4 ± 13.9 kPa (Fig. 2c).

#### Swelling ratio analysis

One of the advantages of gelatin sponge is its high water absorption. Among the four materials, GTA–sponge and GP–sponge exhibit swelling ratios of 956.2% ± 13.1% and 939.5% ± 20.7%, respectively. The swelling rate of mTG–sponge is 1209.3% ± 57.8%, and that of EDC–sponge is the highest, with a value of 1388.3% ± 90.9% (Fig. 2d).

### Hydrolysis properties

**Figure 3:**
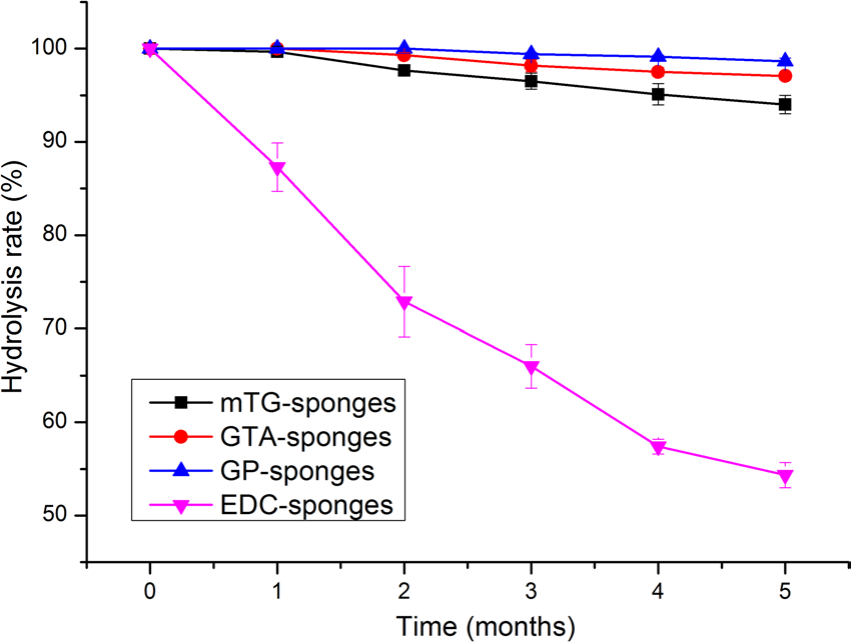
Hydrolysis properties of gelatin sponges prepared by different crosslinking methods.

Un-crosslinked gelatin sponge exhibits poor hydrolytic resistance; for example, 100 mg of un-crosslinked gelatin sponge was completely dissolved in approximately 2 min after being immersed in water. After being crosslinked, the gelatin sponge exhibits significantly enhanced hydrolytic resistance. mTG–, GTA–, and GP–sponges possess extremely high capacity for hydrolytic resistance and had no mass loss in the first month of testing. After 5 months of immersion, all of the three kinds of sponges retained 94% of their original mass. GP–sponge showed the highest remaining mass percentage of about 98%. EDC–sponge presents poor anti-hydrolysis ability. In the first month of testing, the average masses of EDC–sponge decreased to 87.3% of the original mass; the sponge showed a nearly linear decrease in the hydrolysis curve. At the end of the fifth month, the remaining mass percentage is about 54.3% (Fig. 3).

### Enzymolytic properties

**Figure 4:**
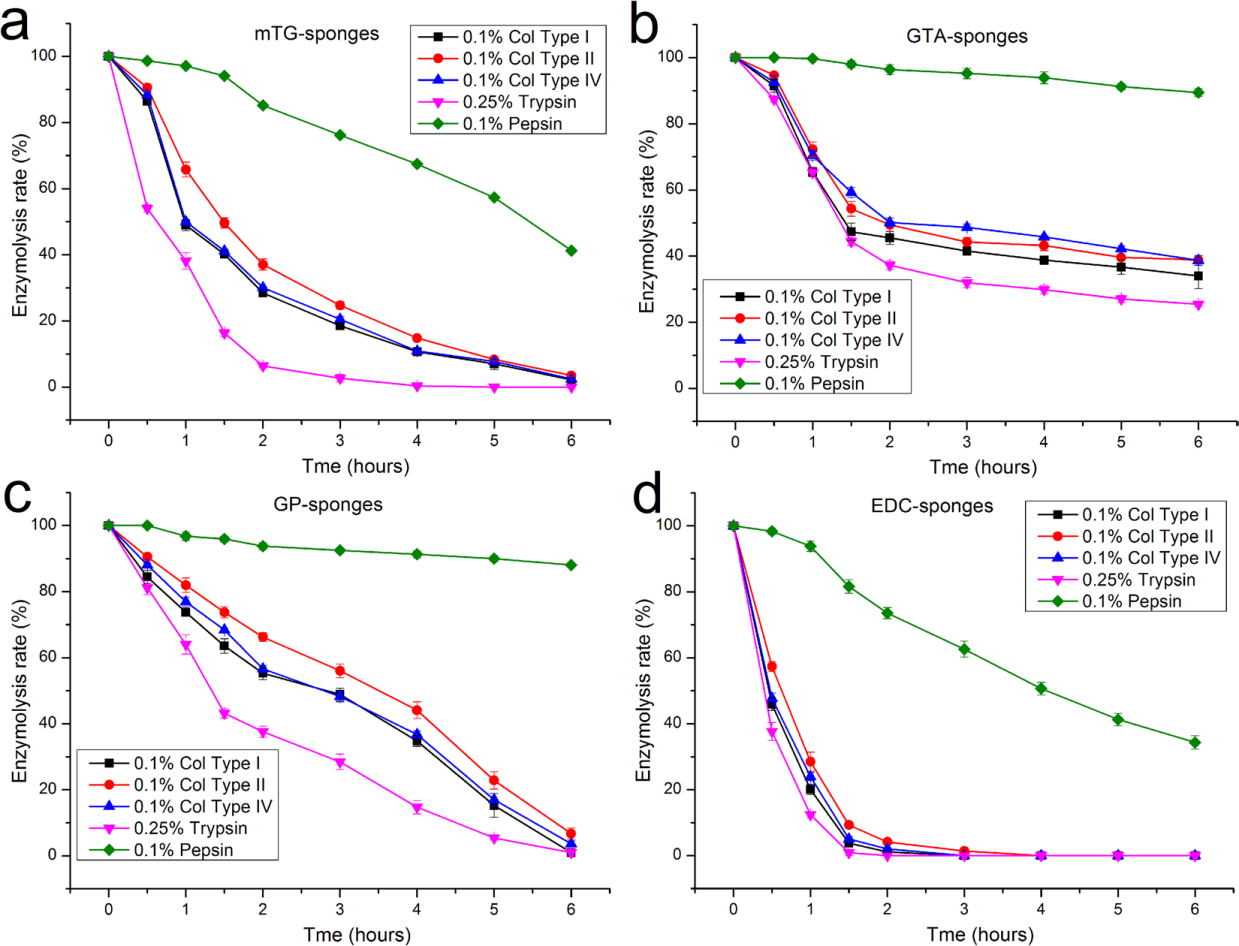
Enzymolytic properties of gelatin sponges prepared by different crosslinking methods. (**a**) mTG–sponge; (**b**) GTA–sponge; (**c**) GP–sponge; and (**d**) EDC–sponge.

Different enzymes were used in the material enzymolysis tests to understand the enzymolytic properties of the gelatin sponges (Fig. 4). Collagenase types I, II, and IV and trypsin and pepsin commonly exist in mammals and are often used in cell culture. The material degradation process of these proteases must be evaluated to provide a basis for cellular inoculation and digestion on gelatin sponges.

Collagenases of types I, type II, and type IV at a concentration of 0.1% are typically used in cellular digestion and isolation from tissues. Despite their differences in subtype classification, the three kinds of collagenases exhibit similar enzymolytic processes to each kind of gelatin sponges. However, the curves of enzymatic degradation vary among different materials. During degradation by collagenases, the mass of EDC–sponge decreased exponentially. After 30 min, the residual mass of EDC is approximately half of the initial mass. About 20% of the original mass remained after 1 h, and about 10% left at 1.5 h. The sponge almost completely dissolved after 2 h of enzymatic degradation. For mTG–sponge, the speed of enzymatic degradation by collagenases is slightly slower than that of EDC–sponge. About 50% mass remained after 1 h of digestion. A residual mass of less than 20% was achieved after 4 h. The enzymolysis rate of collagenase on GP–sponge is markedly slower than those of mTG–sponge and EDC–sponge. The degradation curve showed a slow linear decline. Complete degradation of the material required 6 h. The enzymolysis curves of collagenase on GTA–sponge showed a slightly exponential decline. The first hour of collagenase treatment resulted in degradation to about 70%; in the second hour, the mass of remaining sponge is about 50%. After a slight further decline, the final mass in the sixth hour remained at approximately 40%.

Trypsin solution (0.25%) is commonly used in cellular digestion and passage to dissociate cells from the Petri dish. Based on the enzymolysis tests, 0.25% trypsin on various gelatin sponge shows similar digestive curves but more severe degradation than collagenase. The digestion of gelatin sponges by pepsin was relatively weak. After 6 h of pepsin digestion, EDC–sponge retained about 35% the undigested material. For mTG–sponge, more than 40% of the material was undigested. The GP–sponge and GTA–sponge showed markedly lower pepsin digestion rate, with approximately 90% of the material remaining after digestion for 6 h.

### Subcutaneous implantation

**Figure 5:**
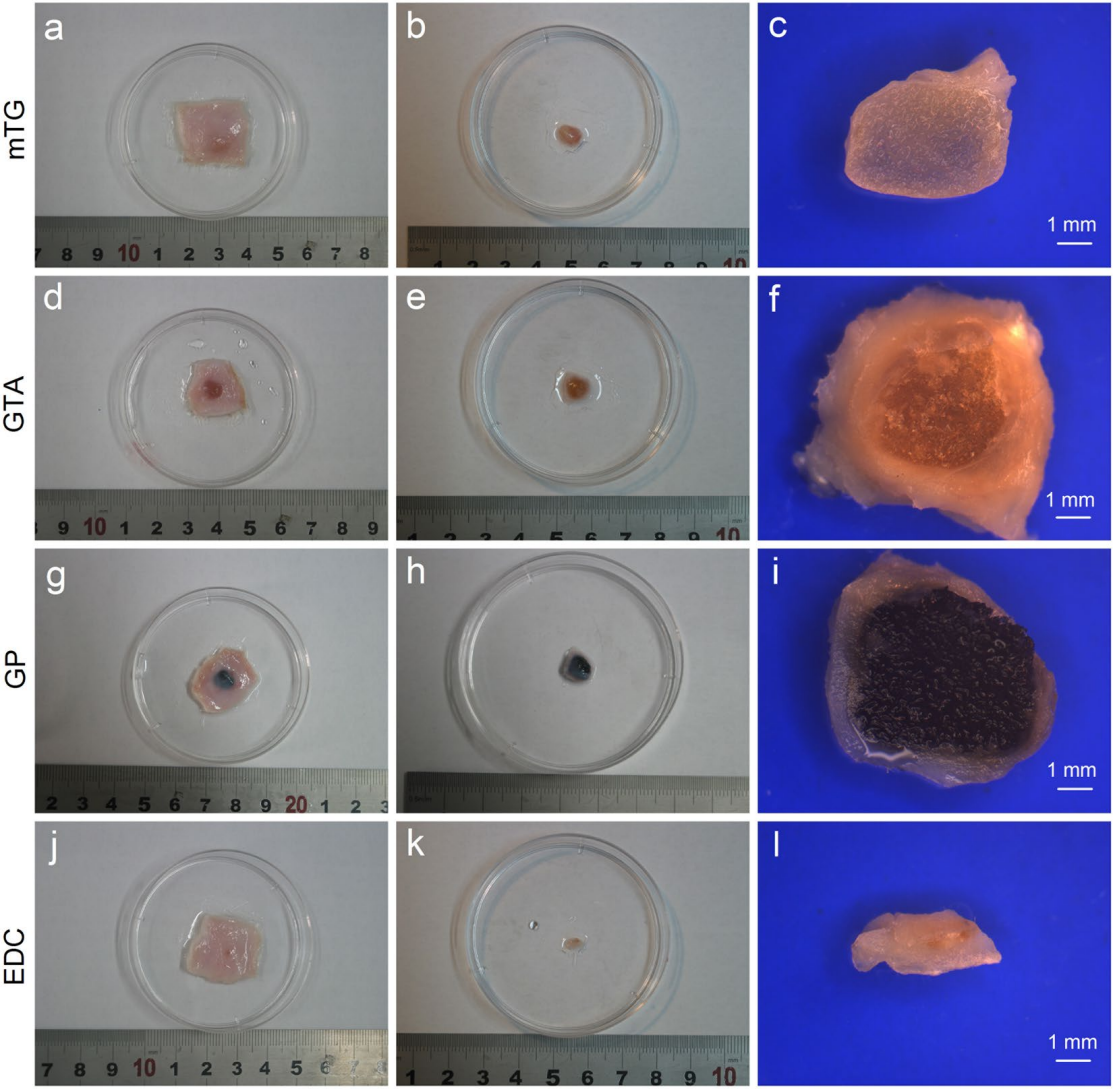
Photographs of gelatin sponges implanted subcutaneously in rats. (**a**,**d**,**g** and **j**) Gelatin sponges with attached skin cut down from the experimental rats after one month subcutaneous implantation; (**b**,**e**,**h** and **k**) gelatin sponges without attached skin; (**c**,**f**,**i** and **l**) cross section of harvested gelatin sponges observed via a stereoscopic microscope. Scale bar = 1 mm.

All experimental rats survived the surgical procedure without surgical complications. The rats were sacrificed 1 month after the operation. The size and shape of the harvested implants differ among the samples. On average, the volume of harvested implants formed by GTA–sponge is higher than those by the other materials. The representative harvested implants are shown in Fig. 5. Further measurements showed that the GTA implant presented the lowest degradation rate, with a remaining volume of 69.1% ± 4.3%. For GP–sponge and mTG–sponge, the residual volumes of the material are 52.8% ± 3.5% and 27.7% ± 2.7%, respectively. EDC–sponge showed the highest degradation rate, with a remaining volume of 2.7% ± 1.7%.

The average thicknesses of the surrounding encapsulating tissue in mTG–, GTA–, GP–, and EDC–implants are 0.19 ± 0.16, 0.85 ± 0.34, 0.51 ± 0.21, and 0.59 ± 0.37 mm, respectively (Fig. 5). In addition, several capillaries were observed growing through the coating tissue into the edge of the mTG–sponges, showing that the mTG–sponge can potentially promote capillary network growth.

### Cellular inoculation and digestion efficiency

#### Cellular inoculation efficiency

**Figure 6:**
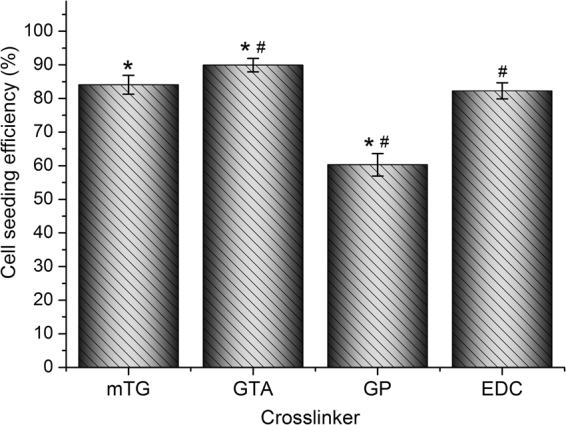
Cellular inoculation efficiency on various crosslinked gelatin sponges. ^*,#^*P* < 0.05.

The cellular inoculation efficiencies on mTG–, GTA–, and EDC–sponges were 84.1 ± 2.8%, 89.9 ± 2.0%, and 82.3 ± 2.4%, respectively. Due to the larger pore size of GP–sponge, the inoculated cells easily leak out of the sponge. As a result of cell leakage, inoculation efficiency decreased to 60.3 ± 3.4% (Fig. 6).

#### Cellular digestion efficiency

**Figure 7:**
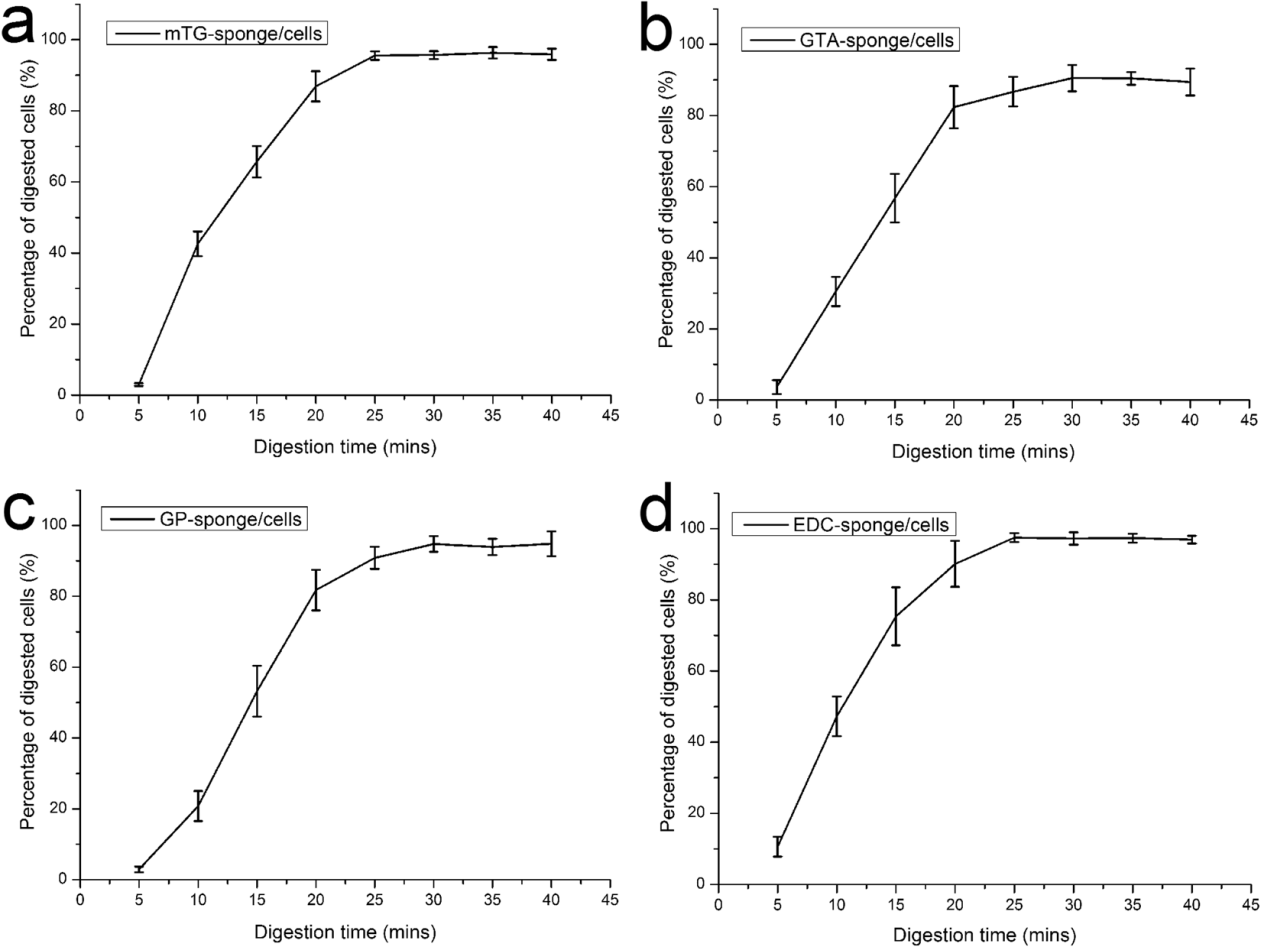
Cellular digestion efficiency assessment on various crosslinked gelatin sponges. (**a**) mTG–sponge/cells construct; (**b**) GTA–sponge/cells construct; (**c**) GP–sponge/cells construct; and (**d**) EDC–sponge/cells construct.

An understanding of the cellular digestion process from sponges will benefit further cell manipulation and analysis, such as cell culture and gene phenotype test, among others. In 2D culture, 0.25% trypsin solution was regularly used to digest the cells from the surface of culture dish, and the digestion time is generally limited to 3–5 min. Cells grown in 3D scaffolds often require a longer digestion time to detach from material surface; however, longer trypsin digestion time results in lower cellular survival rate. As an alternative, 0.1% collagenase type I was used to digest cells from the sponges, and cell digestion efficiency at each time point were calculated. The results show that the digestion efficiency of the four materials was about 90% after 30 min of digestion. With further increase in digestion time, no evident changes in digestive efficiency were observed, as shown in Fig. 7. Therefore, the 30 min digestion time was used as a reference in the subsequent experiments.

### Cellular proliferation in sponges

**Figure 8:**
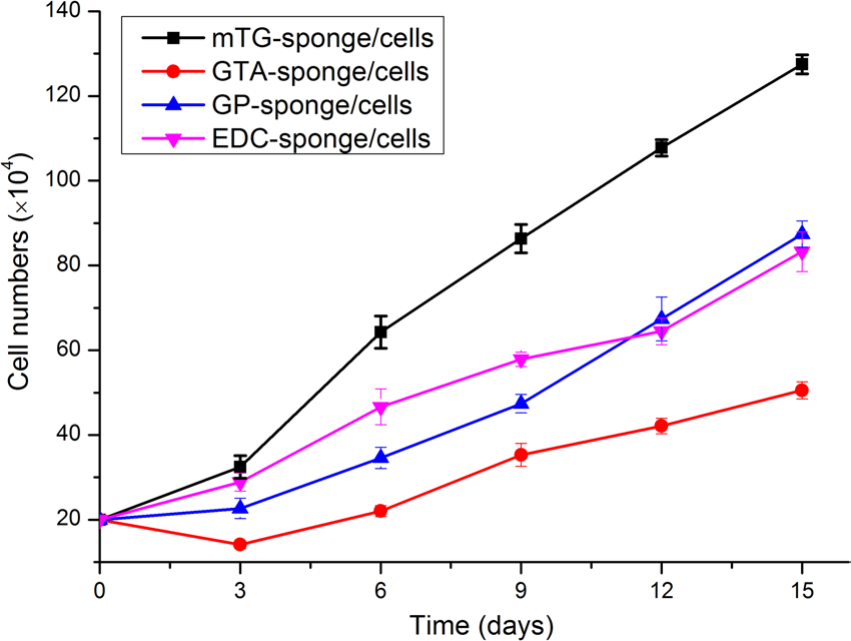
Cell proliferation curves of various crosslinked gelatin sponges.

Cellular growth on various gelatin sponges was tested via MTT cellular proliferation assay. The assay reflected the metabolic activity of mitochondrial dehydrogenase in living cells that transforms light yellow MTT into dark blue formazan^15^. Although all of the materials were inoculated with the same number of cells, the cells grew faster in mTG–sponge with the prolongation of culture time. At each time point, mTG–sponge exhibited a higher cellular proliferation rate than the other materials. The cells grew slowly on the GTA–sponge, and the number of cells was always the lowest among the groups. On the third day, several cells may be affected by GTA toxicity, and the number of cells slightly decreased. Subsequently, with the accommodation of the cells into the GTA–sponge, the cell proliferation curve gradually increased. The cell growth curves of GP–sponge and EDC–sponge are located in the middle of the curves of the above two materials, showing a linear growth pattern. In the 12th day of cell culture, the EDC–sponge showed a slight degradation, and small pieces of material fallen from the sponge led to a reduction in the number of cells (Fig. 8). Furthermore, the statistical analysis has been done at any given days (day 3, 6, 9, 12, 15) among the crosslinked samples. There are no significant differences of cell viability in the group of mTG-sponge and EDC-sponge at day 3 (P > 0.05), and in the groups of GTA-sponge and GP-sponge at day 12 (P > 0.05) and at day 15 (P > 0.05). Except for the above three groups, there are significant differences (P < 0.01) in every random-paired group among the four kinds of crosslinked samples at any given days.

### Cellular live/death analysis

**Figure 9:**
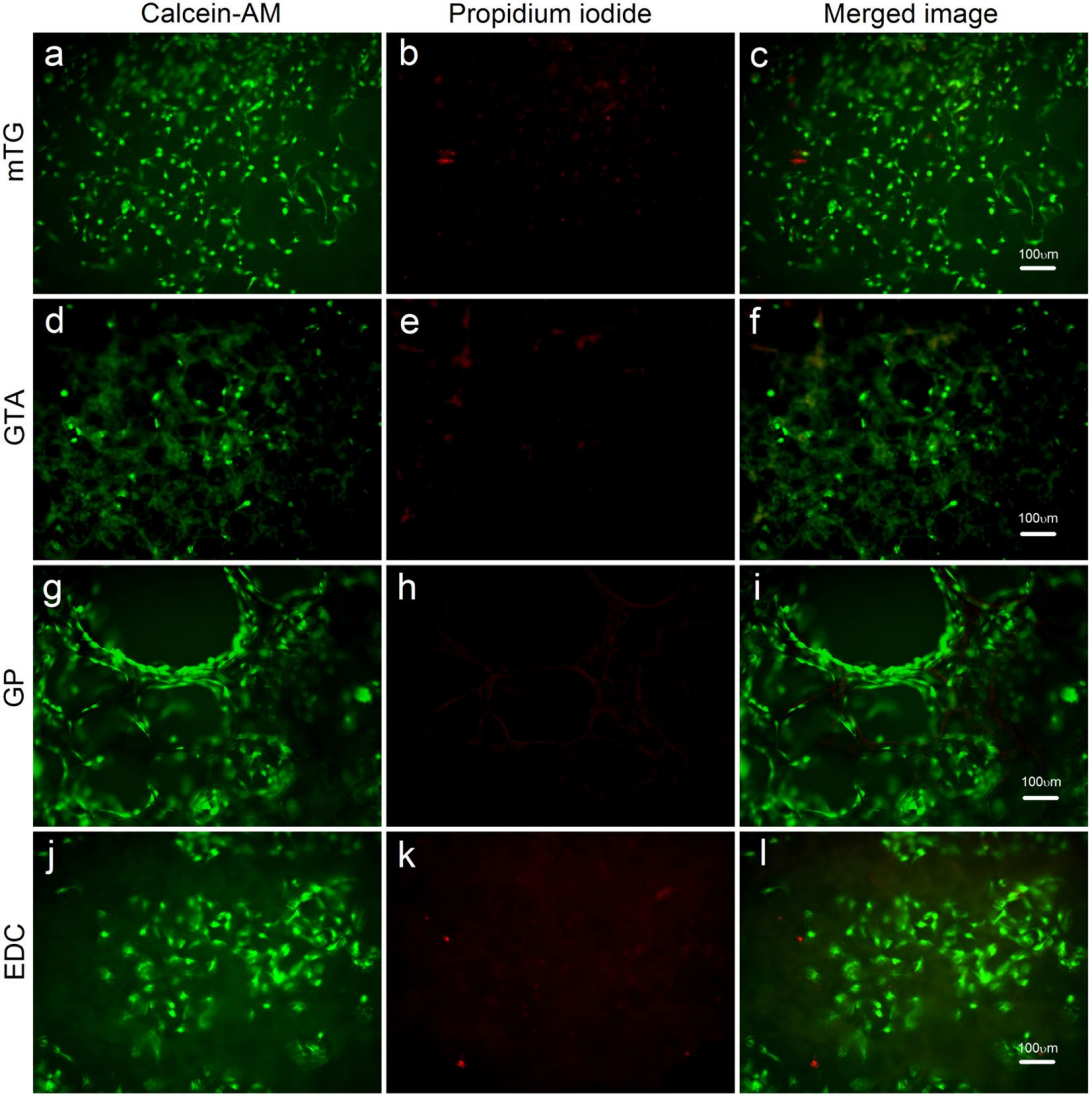
Cellular live/dead fluorescence staining of various crosslinked gelatin sponges. (**a**,**d**,**g** and **j**) Live cells are stained with calcein–AM (green); (**b**,**e**,**h** and **k**) dead cells are stained with propidium iodide (red); and (**c**,**f**,**i** and **l**) merged fluorescence images of cellular live/dead staining. Scale bar = 100 μm.

In the cellular live/death assay, all of the sponge scaffolds exhibited good cellular compatibility. The overwhelming majority of cells were alive. Cellular distribution in mTG–sponge is uniform with a higher cellular survival rate. In comparison, fewer cells were observed in GTA–sponge. Among the four materials, GP–sponge showed a different cell distribution that numerous cells grew around the hole, forming several tubular structures. Given the larger pore size of GP–sponge, several cells penetrated into and grew inside the pores. The cells grew well in EDC–sponge, forming a number of cellular clusters, as shown in Fig. 9.

## Discussion

In tissue engineering, a porous scaffold is required to function as a 3D template and guide in cell adhesion, extension, proliferation, and differentiation. Being a partial hydrolysis product of collagen, the porous gelatin sponge scaffold is theoretically suitable for tissue engineering application. However, the application of pure gelatin sponge in cell culture is extremely limited to date mainly because the gelatin material easily dissolved in aqueous solution, resulting in low tensile strength, easy deformation, and degradation. This problem can be solved by improvement through material crosslinking.

In the current literature, the most commonly used crosslinking agent for gelatin sponge is GTA vapor or GTA solution^15,16,26^. The use of GTA vapor to crosslink materials is time consuming and limited by the thickness of the sponge material because GTA vapor cannot easily penetrate into the interior of the material, resulting in a poor crosslinking degree. The crosslinking reaction by GTA solution is markedly faster than that by GTA vapor, with good crosslinking effect, but the residual GTA is difficult to completely remove after crosslinking. Due to the cytotoxicity of GTA, the residue can affect cell growth. So as to reduce the toxic and side effects of the residual GTA after crosslinking, the GTA crosslinked material is usually treated with glycine solution to inactivate the aldehyde group of the residual GTA. However, in the later washing process of the material, the glycine molecules may fall off, resulting in the exposure of GTA aldehyde group, which will still cause damage to cells.

GP is a naturally occurring crosslinking agent and is superior to other chemical crosslinking agents because of its 10000 x lower toxicity than aldehyde and epoxy crosslinkers^27^. The GP-crosslinked gelatin material is dark blue, so GP is often used as a dye. The GP-crosslinked gelatin material possesses large aperture and high porosity. In this study, the cells grew around the pores of the GP–sponge and assume a tubular shape, which may contribute to the formation of capillaries in the future. Given the relatively large porosity of GP–sponge, nutrient metabolism is more likely to occur, so cells can enter into the interior of the material. Moreover, GP–sponge exhibits good resistance to hydrolysis and enzymolysis.

EDC exhibits biocompatibility and low toxicity because it is not incorporated into the crosslinked sponge structure. As a crosslinker, EDC is widely used in various amino acid-based biomaterials^2,28–30^. However, we found that the hydrolysis resistance of EDC is not ideal, leading to low mechanical strength and ultimately affecting cell growth.

Transglutaminase is a kind of protease that is widely found in mammals, whereas mTG is a homologous protease expressed in microorganisms^31–33^. To date, mTG-crosslinked gelatin sponges have yet to be reported. We hoped that the approach presented in this article may provide a guide for future research. In the preparation of gelatin sponge, we found that a freeze-dried sponge scaffold with uniform pore size is difficult to prepare by using PBS, possibly because the phosphate in PBS affects the formation of ice crystals during the freezing process. Therefore, in the next stage of freeze-drying, the collapse of the sponge scaffold structure will lead to the failure of preparing gelatin sponge. After repeated experiments, we found that use of distilled water to prepare gelatin solution can solve this problem. In addition, more uniform pore size can be obtained by controlling freezing rate during the scaffold synthesis process. Readers can refer to the relevant literature^34^.

Several key problems in the preparation of gelatin sponge must be addressed: (1) concentration of gelatin solution: we currently use 4% gelatin solution, but other concentrations (2–10%) can also be used to prepare sponge material. (2) Concentration of crosslinking agent: for the dosage of mTG, we conducted several discussions in the published article^25^. The key point is to ensure that the material is fully crosslinked. The usual dosage in this study is 10 U mTG per gram of gelatin. The usage of other crosslinking agents, such as GTA, GP, and EDC, was determined by referring to the literature^15,35–37^ and our own practice. For comparison, the concentrations of crosslinkers used in this study are converted to percentage concentrations and molar concentrations, respectively, The percentage concentration of mTG/GTA/EDC/GP solution is about 0.4%/0.5%/0.48%/0.34%, respectively. It can be seen that the percentage of the four crosslinking agents is relatively close. The molar concentration of mTG/GTA/EDC/GP solution is 0.1 mM/50 mM/25 mM/15 mM, respectively. It can be seen that the molar concentration of mTG is very low. mTG is usually composed of 331 amino acids and has a molecular weight of about 38 kDa^31^, far greater than the molecular weight of the other three chemical crosslinking agents. Therefore, the calculated molar concentration of mTG is very low. However, by enzyme catalysis, the gelatin solution can be crosslinked effectively even at lower mTG molar concentration. As for the other three chemical crosslinkers, there is a big difference in the molar concentration reported in the literature. It is still necessary for the experimenters to determine the concentration of the crosslinker according to their own experiments. Furthermore, Chiou *et al*. reported that the gel strength of GP–crosslinked porcine gelatin hydrogels was about 4.5 times that of GTA–crosslinked hydrogels when GP and GTA were used at the same molar concentration (22 mM or 44 mM)^38^. Hence, we speculate that even at relatively low GP molar concentration (15 mM), the strength of the GP-crosslinked gelatin hydrogel may be higher than that by 50 mM GTA. Our experimental results confirm this point. (3) Sterilization of materials: in this study, we used 75% alcohol to sterilize the freeze-dried sponges, followed by half an hour of UV sterilization and the addition of antibiotics to the culture medium, this treatment essentially inhibited the growth of bacteria. Of course, other sterilization methods may also be feasible, such as radiation sterilization or low temperature hydrogen peroxide sterilization.

The mechanical structure of the gelatin sponge is strengthened after crosslinking. In both dry and wet states, the compressive moduli of GTA–sponge and GP–sponge are higher than that of the other two materials. Notably, despite the larger pore diameter and higher porosity, GP–sponge possesses the highest compression modulus among all tested materials, indicating that GP offers extremely strong crosslinking of gelatin molecules. In general, a highly crosslinked polymer exhibits a less degree of swelling, therefore, the swelling rate of GP–sponge is the smallest among the four tested materials. It is worth noting that the swelling rate of sponge scaffolds is not as low as possible. A lower swelling rate means low water absorption and high material hardness, which may be suitable for bone repair, but not suitable for soft tissue repair. In addition, changing the concentration of gelatin solution or crosslinking agent may change the swelling rate of sponges^2,15,37^. The hydrolysis experiments are conducted mainly to test whether the materials will dissolve in the aqueous solution. Except for the EDC–sponge, other materials are highly resistant to hydrolysis. After five months of hydrolysis test, only less than 6% of the mass loss occurred. The hydrolysis property of EDC–sponge was poor but was markedly superior to that of the un-crosslinked gelatin sponge.

Enzymolysis is another important index used to evaluate the quality of scaffold materials, given that the scaffold will be degraded by various enzymes *in vivo* once implanted. If the scaffold is degraded too rapidly, the adhesion of cells and the formation of new tissues will be destroyed. In this study, we used a variety of proteases to test the four kinds of gelatin sponges to understand their enzymolytic properties. The results show that under protease decomposition, most of the materials exhibit different degrees of degradation in a short period of time.

The results of enzymatic testing help us to control the time required for material digestion. In cellular 3D culture, cells are sometimes necessary to be digested from the scaffold for cell subculture or RNA detection. Although trypsin is the most commonly used enzyme in 2D culture, we found that the digestion effect by trypsin is detrimental for the 3D culture because of the expected prolonged digestion time. Considering that extended trypsin digestion time will decrease cell survival rate, we used collagenase for cell digestion. We found that cells could be digested from the sponges by various collagenases within 30 min, while the material only showed a small amount of degradation. The four kinds of gelatin sponges showed weak digestion by pepsin; thus, pepsin is unsuitable for use as a digestive solution. Moreover, given that collagenase and trypsin markedly affect the degradation of the gelatin sponges, the enzymes should be removed from the cellular seeding solution to the greatest possible extent when the sponges are inoculated with cells.

Subcutaneous implantation is a common way to evaluate material biocompatibility^39–41^. In the subcutaneous implantation experiment, GTA–sponge and GP–sponge were the most resistant to material degradation. However, both materials showed obvious tissue rejection and thicker coating tissue. It may be due to the friction between hard implanted material and subcutaneous tissue, and the inflammatory reaction caused by the residual chemical crosslinkers presenting in the implanted materials. In contrast, mTG–sponge presents a thin coating layer, which indicates small tissue rejection. This is mainly due to the advantage of enzymatic crosslinking. It has been documented that residual mTG does not cause damage to animal tissues or cells^24,32,42^. Furthermore, mTG-sponge has good water absorption and softness, it reduces the friction in subcutaneous tissue and leads to a thinner coating layer. In addition, several blood vessels are seen on the outer coating of the implanted material. The formation of capillaries in mTG–sponge is beneficial to the growth of new tissue. EDC–sponge is more likely to degrade^28^, resulting in minimal residual mass after implantation; however, its coating layer is thicker than that of mTG–sponge.

ADSCs, which present some of the most promising adult stem cells, are derived from adipose tissue. Since the cells were first characterized by Zuk *et al*.^43^ in 2001, ADSCs have been extensively studied for their multiple differentiation potentials, including osteogenesis, adipogenesis, chondrogenesis, and cardiogenesis^44,45^. ADSCs can be isolated easily with minimally invasive surgery on donors through lipoaspiration under local anesthesia, and larger amounts of ADSCs can be obtained^46^. ADSCs can be subcultured for more than 20 passages without losing their stem cell characteristics in the expression of reprogramming transcriptional factors^47^. In the cell proliferation tests, the best growth of cells was observed on mTG among all materials. At each of the time points, cellular proliferation rate on mTG–sponge was higher than that in the other test groups. The cells on GTA–sponge grew slowly possibly because GTA toxicity. The cell growth curve of GP–sponge is located in the middle of the curves of the above two materials and show a linear growth pattern. The number of cells in EDC was the lowest mainly because the material shows certain degradation and swelling, which result in a relatively small number of cells

In conclusion, we propose several methods for preparing gelatin sponges and made a detailed comparison to understand their physical characteristics and biocompatibility. Among the four tested gelatin sponges, GTA material exhibits certain cytotoxicity, and EDC shows a rapid degradation rate. The GP material is hard, and strong tissue rejection is observed after subcutaneous implantation. mTG–sponge exhibits the best comprehensive performance, with good porosity, compressive modulus, and anti-degradation capacity. Moreover, the material presents good *in vitro* cell compatibility and *in vivo* biocompatibility. Hence, we recommend that the mTG –sponge is a suitable biomaterial for most soft tissue repair, as it has good water absorption, flexibility and biocompatibility. The GP–sponge is a suitabe biomaterial for hard tissue repair, as it has lower water absorption, larger pore size and higher mechanical strength.

## Materials and Methods

### Preparation of gelatin sponges through different crosslinking methods

#### mTG-crosslinked gelatin sponge (mTG–sponge)

mTG-crosslinked gelatin hydrogel was prepared using a protocol described in our previous publication^25^. Briefly, gelatin (type A, 300 Bloom; Sigma, USA) was dissolved in deionized water at 50 °C to obtain 4% solution and sterilized through 0.22 μm filter. mTG (Bomei, China, enzyme activity >100 U per gram) was dissolved in phosphate-buffered saline (PBS) to obtain 10% solution and sterilized through 0.22 μm filter. Gelatin/mTG hydrogel was obtained by mixing gelatin and mTG solution at a ratio of 40 μL of mTG per milliliter gelatin solution. The sample was incubated at 37 °C until gelling. The resultant hydrogel was frozen at −20 °C for 8 h and lyophilized for 48 h to become a sponge.

#### GTA-crosslinked gelatin sponge (GTA–sponge)

25% GTA solution was added to the 4% sterilized gelatin solution to achieve a final 0.5% (v/v) GTA concentration. The mixed solution was incubated at 37 °C until gelling. The crosslinked sponges were placed in 100 mM glycine solution at room temperature for 2 h to block the residual aldehyde groups of GTA. The hydrogels were washed with deionized water, frozen at −20 °C for 8 h, and lyophilized for 48 h.

#### GP-crosslinked gelatin sponge (GP–sponge)

GP powder (Zhixin Bio-technology, Jiangxi, China) was dissolved in 70% ethanol to obtain 300 mM stock solution. GP-crosslinked gelatin hydrogels were prepared by adding the GP stock solution to the 4% sterilized gelatin solution to achieve a final GP concentration of 15 mM. The mixed solution was incubated at 37 °C until gelling. The resultant hydrogels were frozen at −20 °C for 8 h and lyophilized for 48 h.

#### EDC-crosslinked gelatin sponge (EDC–sponge)

EDC (Oddfoni, Jiangsu, China) and NHS (*n*-hydroxysuccinimide, Oddfoni, Jiangsu, China) were dissolved in deionized water to obtain 1 and 0.4 M stock solutions, respectively. Both solutions were sterilized through 0.22 μm filters. EDC/NHS-crosslinked gelatin hydrogels were prepared by adding EDC and NHS stock solutions to the 4% sterilized gelatin solution to achieve 25 mM EDC and 10 mM NHS. The solutions were then incubated at 37 °C until gelling. The resultant hydrogels were frozen at −20 °C for 8 h and lyophilized for 48 h.

#### Un-crosslinked gelatin sponge

Un-crosslinked gelatin sponge was prepared by pre-cooling the 4% gelatin solution at 4 °C for 2 h, freezing at −20 °C for 8 h, and lyophilizing for 48 h. The sponges were served as control groups in the following experiments.

The appearances of the prepared sponges in dry state and wet state (after immersion in PBS for 1 h at 37 °C) were observed under a stereoscopic microscope (Jinteng, China) equipped with a digital camera (MD50, Mingmei, China).

### Porosity measurement

The porosity of the gelatin sponges was evaluated through liquid displacement method^48^. Absolute ethanol was used as displacement liquid because it permeates through the sponges without inducing matrix swelling or shrinkage. A pre-weighed lyophilized sponge was immersed into a known volume (*V*_1_) of ethanol and degassed for 5 min with a vacuum pump. The total volume of ethanol and ethanol-impregnated sponge was recorded as *V*_2_. The ethanol-impregnated sponge was removed, and the residual volume of ethanol was recorded as *V*_3_. The porosity (ε_1_) of the sponge was calculated as follows:

1 $${\varepsilon }_{1}( \% )=({V}_{1}-{V}_{3})/({V}_{2}-{V}_{3})\times 100$$

### Mechanical testing

The compressive moduli of dry and wet gelatin sponges were systematically investigated on a uniaxial mechanical testing apparatus (HPB, Handpi, China) equipped with 20 N capacity. Wet sponges were obtained by soaking the dry sponges in PBS for 1 h. Cylinder-shaped samples with 10 mm diameter and 5 mm height were prepared from the dry and wet sponges prior to testing. Only samples with flat surfaces were selected as test specimens. The samples were placed on a thermostat aluminum stage (DB-H, Xinbao, China) at a constant temperature of 37 °C. A solid compression plate was used for testing, and a small tare load of 0.01 N was applied to ensure that each sample received the same degree of compression. The samples were tested to failure at a crosshead speed of 1 mm/min. Data on stress versus strain were obtained from load and displacement measurements. Compressive modulus was determined from the slope of the linear portion of the stress–strain curve.

### Swelling ratio and hydrolysis property

#### Swelling ratio

A pre-weighed lyophilized sponge (*W*_0_) was immersed into deionized water at room temperature for 1 h. The swelling sponge was obtained. Excess water on the sponge surface was gently removed with filter paper. The sponge was weighed again (*W*_1_). Swelling ratio (ε_2_) was calculated as follows:

2 $${\varepsilon }_{2}( \% )=({W}_{1}-{W}_{0})/{W}_{0}\times 100$$

#### Hydrolysis property

The hydrolysis property of the crosslinked gelatin sponges was evaluated by immersing them in deionized water for a period of time. Degradation rates of the sponges were then assessed. Pre-weighed lyophilized gelatin sponges derived from different crosslinking methods were sterilized with 75% alcohol for 20 min to avoid biomaterial degradation caused by bacterial contamination. The sponges were then washed several times with sterilized deionized water. The swelling sponges were kept in water and placed in a cell incubator at 37 °C. At the specified time points of 1–5 months, the remaining sponges were obtained, lyophilized, and weighed. The extent of biomaterial hydrolysis was determined by calculating the percentage of remaining weight compared with the original weight.

### *In vitro* enzymatic stability

The biological stability of the gelatin sponges was evaluated by exposing them to various enzymes to assess degradation rates. Wet sponges were prepared by soaking the lyophilized sponges in PBS for 1 h. Pre-weighed wet sponges were exposed to 0.1% collagenase type I (>125 CDU/mg, Invitrogen, CA, USA), 0.1% collagenase type II (>125 CDU/mg, Invitrogen), 0.1% collagenase type IV (>125 CDU/mg, Invitrogen), 0.25% trypsin (250 NFU/mg, Invitrogen), and 0.1% pepsin (800–2500 U/mg, Sigma) for 6 h. All the enzymatic solutions were prepared in PBS. Enzymatic degradation tests were performed at 37 °C in a horizontal shaker. At the specified time points of 0.5, 1, 1.5, 2, 3, 4, 5, and 6 h, the remaining sponges were collected. Excess water was gently removed from the sponge surface by using filter paper. The sponge was weighed again. The extent of biomaterial degradation was determined through calculating the percentage of remaining weight versus the original weight.

### *In vivo* biocompatibility of gelatin sponges

Animal study was approved by the Institutional Animal Care and Use Committee (IACUC) of Sichuan University, all experiments were performed in accordance with the guidelines of IACUC of Sichuan University. *In vivo* biological responses to the gelatin sponges were assessed in subcutaneous Sprague-Dawley (SD) rat models by using the method described in our previous publication^25^. Briefly, four kinds of gelatin sponges were sterilized in 75% ethanol for 20 min, followed by 30 min UV irradiation and several washings with PBS, and equilibrated in DMEM for 12 h. The wet sponges were cut with a scalpel in a sterile bio-safety cabinet into a size of 7 mm × 7 mm × 4 mm. The sponges were surgically placed within subcutaneous pockets located on the dorsum of adult SD rats (age 7–8 weeks). Each of 10 rats received two randomized dorsal subcutaneous implants. Each material had four duplicate samples. The remaining four incisions were sutured directly without implantation and served as negative control. After 1 month of implantation, the rats were sacrificed. The implanted sponges and the surrounding connective tissues and skin were carefully resected from the underlying muscles. Implants with the coating tissue were further dissected from the surrounding skin, and their length/width/heights were measured using a Vernier caliper to estimate the volume of the implants. For material degradation analysis, the harvested implant was cut along the horizontal plane to expose the cross section for imaging. Images were analyzed using an image processing software (Image Pro Plus 6.0, IPP6, Media Cybernetics, MD, USA). The average thickness of the surrounding encapsulating tissue was measured along the cross section by using a straight line tool of IPP6. The volume of the encapsulating tissue was also estimated. The approximated volume of the remaining sponge was calculated by subtracting the volume of the encapsulating tissue from the entire implant volume. The extent of *in vivo* biomaterial degradation was determined through calculating the percentage of the remaining volume versus the original volume of the sponge.

### Cellular inoculation and digestion

#### Cellular seeding efficiency

Primary ADSCs were isolated using a previously described method^25,49^. The four kinds of wet gelatin sponges were prepared as described above. All of the sponges were aspirated dry prior to cell seeding. The dry gelatin sponges were placed in each well of a 24-well plate to collect cells that did not attach to the sponge during cell seeding. Each gelatin sponge was seeded with 200 μL of ADSC suspension (~1.0 × 10^6^ cells). The sponges were removed, and cells in each well were collected from the medium and well. The number of cells was counted using a hemacytometer. The difference between the number of seeded cells and the number of leaked cells was considered as the number of cells that adhered to each sponge. Seeding efficiency was calculated by dividing the number of adhered cells by the number of seeded cells. Five samples were used for each kinds of the sponges to obtain averages and standard deviations.

#### Determine digestion time

As described above in this section, the cells were seeded onto the four kinds of gelatin sponges and transferred to 24-well plates added with cell culture medium. The plates were placed in a cell incubator for 8 h to allow the cells to fully attach to the materials. After incubation, the medium was aspirated. The sponges were rinsed three times in PBS and added to 0.1% collagenase type I for cellular digestion. At various time points (5–40 min, at a 5 min interval), the digestive solutions were collected. Cells were counted using a hemacytometer. Cellular digestion efficiency was calculated by dividing the number of digested cells by the number of adhered cells.

### *In vitro* cell proliferation studies

Cell proliferation was tested by thiazolyl blue assay (MTT, Sigma, USA). Gelatin sponges were prepared as described above. ADSCs were inoculated at 2 × 10^5^ cells per sponge and incubated at 37 °C with 5% CO_2_ in a humidified atmosphere. Cellular growth pattern in the sponge was observed daily via an inverted microscope (CKX41, Olympus, Japan). At each time point (0, 3, 6, 9, 12, and 15 d), samples of cell-containing sponges were washed three times with PBS. Enzymatic digestion was conducted by adding 0.1% collagenase type I to each sponge, followed by incubation at 37 °C for 30 min. Digested cells were collected, centrifuged, washed with PBS, and centrifuged again. The pelleted cells of each sample were resuspended with 800 µL of high-glucose DMEM plus 80 µL of MTT solution (5 mg/mL in PBS). The cells were pipetted into a 24-well plate and incubated for 4 h at 37 °C and 5% CO_2_. The supernatant was removed after incubation, and the formazan crystals in the cells were solubilized by adding 400 µL of DMSO to each sample. MTT reduction was quantified by recording light absorbance by using a microplate reader (Biotek ELx800, USA) at 490 nm with a reference wavelength of 630 nm. Background absorbance from control wells (medium without cells) was subtracted. The assays were performed in triplicate for each experimental condition.

### Cell viability

ADSC viability in various gelatin sponges was assessed via live/dead-staining assay. Cell-containing sponges were prepared as mentioned above. After 2-week culture, cell-containing sponges were washed in sterile PBS and cut with a scalpel in a sterile bio-safety cabinet. The slices were cut at 1 mm intervals along the transverse planes of the sponge. Representative slices from the center of the sponge were incubated at 37 °C for 30 min in a solution containing 4 μM calcein–AM (Sigma, MO, USA) and 4 μM propidium iodide (Sigma, MO, USA) in PBS. After incubation, the samples were washed again and viewed under an inverted fluorescent microscope (XDS30, Sunny, China) equipped with a digital camera.

### Statistical analysis

Data are presented as mean ± SD. Statistical analyses were performed using SPSS software (version 14.0). Statistical significance was evaluated using one-way ANOVA with least significant difference test. The level of statistical significance was set at *P* < 0.05.

## Electronic supplementary material

Dataset1
